# Low-temperature synthesis of 2D anisotropic MoTe_2_ using a high-pressure soft sputtering technique†

**DOI:** 10.1039/d0na00066c

**Published:** 2020-03-04

**Authors:** Kentaro Yumigeta, Cameron Kopas, Mark Blei, Debarati Hajra, Yuxia Shen, Dipesh Trivedi, Pranvera Kolari, Nathan Newman, Sefaattin Tongay

**Affiliations:** School for Engineering of Matter, Transport and Energy, Arizona State University Tempe AZ 85287 USA sefaattintongay@asu.edu

## Abstract

We demonstrate a high-pressure soft sputtering technique that can grow large area 1T′ phase MoTe_2_ sheets on HOPG and Al_2_O_3_ substrates at temperatures as low as 300 °C. The results show that a single Mo/Te co-sputtering step on heated substrates produces highly defected films as a result of the low Te sticking coefficient. The stoichiometry is significantly improved when a 2-step technique is used, which first co-sputters Mo and Te onto an unheated substrate and then anneals the deposited material to crystalize it into 1T′ phase MoTe_2_. A MoTe_2−*x*_ 1T′ film with the lowest Te vacancy content (*x* = 0.14) was synthesized using a 300 °C annealing step, but a higher processing temperature was prohibited due to MoTe_2_ decomposition with an activation energy of 80.7 kJ mol^−1^. However, additional *ex situ* thermal processing at ∼1 torr tellurium pressure can further reduce the Te-vacancy (V_Te_) concentration, resulting in an improvement in the composition from MoTe_1.86_ to MoTe_1.9_. Hall measurements indicate that the films produced with the 2-step *in situ* process are n-type with a carrier concentration of 4.6 × 10^14^ cm^−2^ per layer, presumably from the large V_Te_ concentration stabilizing the 1T′ over the 2H phase. Our findings (a) demonstrate that large scale synthesis of tellurium based vdW materials is possible using industrial growth and processing techniques and (b) accentuate the challenges in producing stoichiometric MoTe_2_ thin films.

## Abstract 2

High-pressure soft sputtering: large area 1T′ phase MoTe_2_ thin films were grown at temperatures as low as 300 °C.

## Graphical abstract

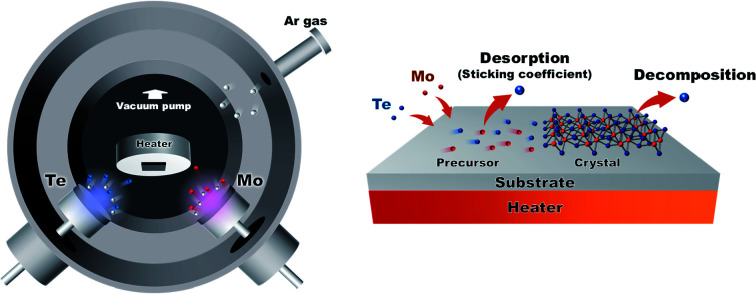

## Introduction

Two-dimensional (2D) transition metal dichalcogenides (TMDs) have attracted much attention owing to their unique quantum properties ranging from excitonics^3–5^ to Weyl fermions^6,7^ and to charge density waves.^7–9^ For example, TMDs made from group VIB Mo and W metals, such as MoS_2_ and WSe_2_ are direct gap excitonic semiconductors with strong exciton binding energies as large as 0.5 eV. This allows for the stabilization of excitons even at room temperature.^3,5,10–12^ The K-valley in their band structure is degenerated for different spin due to broken inversion symmetry. This leads to exotic spin-valley coupling and generates exotic valleytronic properties.^13^ While the S and Se containing group-VIB 2D dichalcogenides all naturally crystallize in the hexagonal (2H) phase, when metal cations bind to tellurium, new phases emerge and 1T′ anisotropic and 2H isotropic phases start to compete.^14–17^ WTe_2_ is a 2D Weyl semimetal crystallizing only in the 1T′ phase.^14,18^ In contrast, 2D MoTe_2_ is naturally stable both in the 1T′ type-II Weyl fermion^17^ phase as well as 2H excitonic semiconductor phase. The latter has a 1.1 eV infrared band gap and the largest exciton binding energy (∼580 meV) reported to date.^15,16^ This indicates that the crystalline phases of 2D dichalcogenides have similar formation energies, which result in both new, exciting properties and manufacturing challenges.

Despite their enticing properties, there has been limited progress in producing high quality, large-area MoTe_2_ films. Powder-chemical vapor deposition (CVD) is often adopted for the large scale synthesis of sulfur and selenium based 2D TMDs.^19^ In this method, target substrates are placed close to powder precursors such as MoO_3_, MoCl_5_, WO_3_, *etc.* and heated up to a high temperature in the presence of H_2_S or S_2_ gas.^20^ This method enables the synthesis of MoS_2_, WS_2_, and others,^19,21,22^ but lacks precise control of gas precursor concentrations and flow, as well as film thickness, and defect concentrations, and is thus unsuitable for producing device-quality films needed in large-scale manufacturing. The powder-CVD synthesis of MoTe_2_ requires much higher processing temperatures (∼800 °C) for crystallization to occur. This introduces a large number of tellurium vacancies to a level that the synthesized MoTe_2_ becomes highly unstable in air. This is the reason that CVD MoTe_2_ sheets are not commercially available. Considering these challenges, MoTe_2_ films have been synthesized by transforming MoO_3_ or Mo thin films into MoTe_2_ at temperatures above 700 °C and at high Te vapor pressures. This is commonly known as the chalcogenation process.^23,24^ This process relies on the diffusion and reaction of elemental Te, but typically yields poor crystallinity materials. Since the chalcogenation occurs at a high temperature, it is not compatible with conventional manufacturing methods and cannot produce vdW superlattices, which require low processing temperatures to retain the layer's structural quality.

**Fig. 1:**
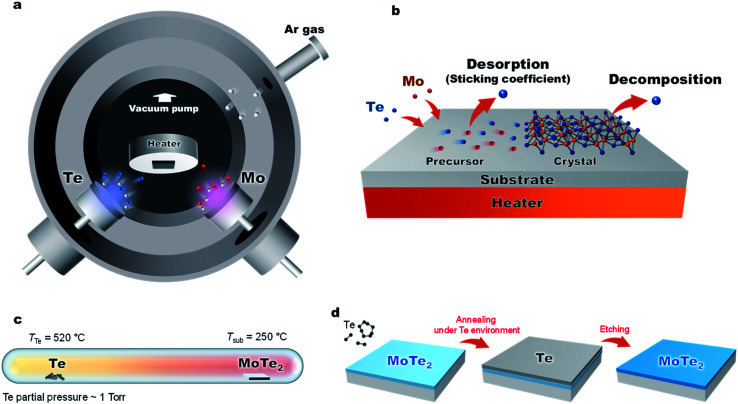
(a) Schematic diagram of the low-energy sputtering deposition. (b) MoTe_2_ growth is limited by a tellurium deficiency due to (1) Te desorption from a precursor state and (2) decomposition of fully-reacted MoTe_*x*_. (c) Diagram of the post annealing process of the MoTe_2_ film under Te pressure. (d) Post thermal process and Te etching process.

In this work, we have developed a low-temperature and low-energy co-sputtering technique (Fig. 1a) to grow MoTe_2_ films over large areas. The sputtering technique allows precise thickness control by adjusting the deposition time and produce thin films at a low processing temperature. By using high pressure growth, the proposed method reduces the kinetic energy of the impinging Mo, Te and Ar atoms to avoid surface and subsurface damage to the MoTe_2_ sheets. To understand what limits the growth conditions of MoTe_2_,^25^ relative sticking coefficients and decomposition rates were determined by co-sputtering Mo and Te as a function of temperature and time (Fig. 1b). To produce enhanced stoichiometric films, Mo and Te atoms were co-deposited onto vdW graphene surfaces at room temperature, followed by *in situ* thermal crystallization at 350–450 °C. We have further improved the stoichiometry from *x* = 1.86 to 1.90 by *ex situ* thermal processing at elevated Te pressures (Fig. 1c and d). The resulting MoTe_2_ shows excellent structural and physical properties, as evidenced by Rutherford backscattering spectroscopy (RBS), Raman spectroscopy, AFM, kelvin probe force microscopy (KPFM), and 4-point resistivity measurements. Interestingly, the deposited MoTe_2_ films always crystallize in a 1T′ type-II Weyl semimetal structure due to large sheet carrier density (originating from V_Te_) driven 1T′ phase stabilization.^1,2^ The overall results optimize a new low-energy sputtering technique that can produce high-quality large-area MoTe_2_ at low temperatures.

Fig. 1a and 3a show the schematic diagram of the deposition chamber and the most-often used temperature timetable, respectively. The ultra-high vacuum (UHV) chamber is evacuated to a low 10^−7^ torr range prior to deposition, followed by sputtering. In our studies, elevated Ar pressures of 150 mTorr were used during the sputtering process to decrease the deposition rate and reduce the kinetic energy of the impinging species on the growth surface. The latter is important to minimize surface and subsurface damage to the growing film, while potentially enhancing surface diffusion. The deposition rate of each element is dependent on sputter power. With increased sputter power of the Te source, we observed effects of charge build-up on the Te target, which caused rather unpredictable deposition rates. To avoid this charge buildup, the Te sputter power was fixed to 5 W while using a DC sputtering source. The effects of the Mo sputter power on the crystal quality and Te/Mo ratio were studied and we optimized the conditions (Fig. S2†).

### Sticking coefficient and decomposition rate

**Fig. 2:**
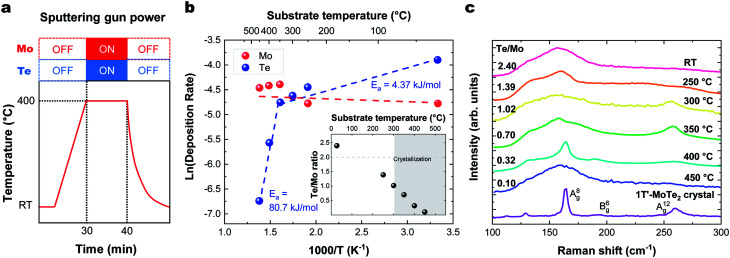
(a) Temperature profile for sticking coefficient measurement. Substrate temperature dependence of (b). the Arrhenius plot of deposition rate *vs.* temperature on (inset) the Te/Mo ratio. Deposition rate is the number of atoms divided by the deposition time (×10^15^ atoms per cm^2^ per s). (c) Raman spectra of MoTe_2_ films deposited at different temperatures.

The sticking coefficient is determined by the adsorption energy of precursor atoms (Mo and Te atoms) on the growth surface. The decomposition rate is determined by the energy of the atoms that are part of the fully reacted MoTe_2_ phase, and is indistinguishable from the evaporation rate of a bulk solid. These two factors ultimately dictate growth characteristics and are two critical parameters for the establishment of a controllable growth process.^26^ To determine these growth parameters and access the Mo and Te sticking coefficients and decomposition rates, the amount of Mo and Te evaporated and deposited was measured during co-sputtering onto graphite/sapphire and bare c-cut sapphire substrates at substrate temperatures ranging from room temperature to 450 °C (Fig. 2a).

The typical thickness of the deposited Mo/Te film was approximately a few nanometers, as determined by Rutherford backscattering spectroscopy (RBS). The number of Mo and Te atoms deposited on sapphire substrates per unit area is measured by RBS, then divided by the growth time to determine the deposition rates. By taking the slope of an Arrhenius plot of the deposition rate, we extracted the activation energy for decomposition, 
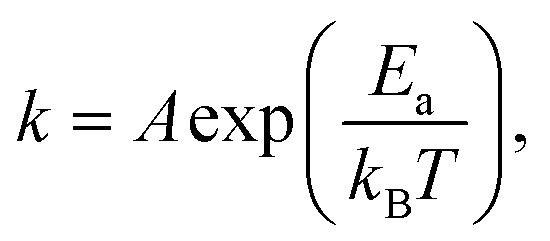
 as shown in Fig. 2b. Closer inspection of this figure reveals two main properties: (1) first, it can be seen that the Mo deposition rate (red circles in Fig. 2b) is nearly independent of the substrate temperature, while the Te deposition rate significantly decreases above 300 °C. Tellurium metal has a high vapor pressure at low temperatures (∼10^−2^ torr at 400 °C), while Mo's is very small (extrapolated to be ∼10^−40^ torr at 400 °C). The normalized Te sticking coefficient, referenced to the room temperature deposition rate, decreases with temperature to a low coefficient of 0.58 at 450 °C. This results in highly Te deficient MoTe_2_ films. (2) The Arrhenius plot for Te shows that there are two distinct slopes with two different activation energies (Fig. 2b blue circles). The lower activation energy (*E*_a_ = 4.3 kJ mol^−1^) is well-defined between 20 °C and 350 °C and is related to the desorption of the precursor forms of tellurium from the growth surface, often referred to as the sticking coefficient. We attribute the higher activation energy (*E*_a_ = 80.7 kJ mol^−1^) to the evaporation of Te as a result of the thermal decomposition of MoTe_2−*x*_.

Raman data collected on these films deposited in a range of temperatures (Fig. 2c) show that crystallization occurs at 300 °C and above, as evidenced by the emergent of out-of-plane A^(8)^_g_ (163 cm^−1^) and A^(12)^_g_ (259 cm^−1^) phonon modes. In-plane B^(6)^_g_ (190 cm^−1^) modes of 1T′-MoTe_2_ were observed at 400 °C. The Raman spectra of the films deposited on HOPG and sapphire showed similar substrate temperature dependence and Raman spectra. These Raman peaks match with the literature values.^27^ This absence of Raman peaks in films deposited below 300 °C and above 450 °C indicates that the associated films do not contain significant levels of crystalline MoTe_2_. For low temperature growth, there is not enough thermal energy available to overcome the kinetic barriers of thin film growth (*e.g.* surface diffusion and reaction). And for high temperature growth, Te-poor films are formed as a result of the high MoTe_*x*_ decomposition rate.

### MoTe_2_ film deposition at room temperature followed by *in situ* annealing

**Fig. 3:**
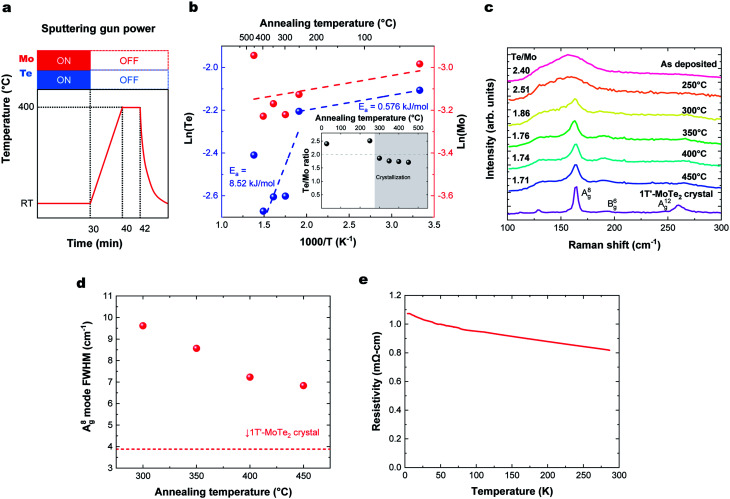
(a) Temperature profile for MoTe_2_ thin film growth followed by *in situ* annealing. (b) Annealing temperature dependence of Te and Mo deposition rates, with (inset) the number of atoms and Te/Mo ratio, where Te and Mo are the number of Te, Mo atoms on the substrate divided by the deposition time (×10^15^ atoms per cm^2^ per s). (c) Raman spectra. (d) Annealing temperature dependence of A^(8)^_g_ Raman peak width. (e) Temperature dependence of resistivity of deposited MoTe_2_.

To produce a film with better stoichiometry, we co-sputtered Mo and Te at room temperature, where the sticking coefficient is high, followed by an *in situ* annealing. The temperature profile of this process and corresponding deposition rate and Te/Mo ratio on the sapphire substrate are shown in Fig. 3a and b, respectively. We note that there were no observable differences in the deposition rate or Te/Mo ratio when MoTe_2_ was deposited onto sapphire and HOPG substrates. Similar to the previous section, our findings reveal that there are two activation energies for Te. One low energy desorption process and another higher energy decomposition process that dominates above 300 °C. The experiments support the following observations: (1) Mo and Te atoms deposited at room temperature have a sticking coefficient near 1 and (2) annealing of the composite in the range of 300–350 °C provides sufficient energy to facilitate a reaction, without experiencing a high Te desorption rate. This allows us to produce MoTe_2−*x*_ thin films with a high Te/Mo ratio (*x* < 0.3).

The degree of crystallization of 1T′ MoTe_2_ can best be monitored by observing the temperature-dependence of the Raman spectrum above 300 °C (Fig. 3c). Raman spectra of the films for HOPG and sapphire showed a similar substrate temperature dependence and Raman spectra (Fig. S2†). The minimum crystallization temperature of ∼300 °C is similar to that found when Mo and Te are sputtered onto heated substrates, as described above. The Raman peak width represents a gauge of the crystallinity of the films, as defects and grain boundaries create local strain and broaden Raman peaks. The crystallinity of the films as a function of annealing temperature was analyzed using the figure-of-merit of FWHM of the A^(8)^_g_ mode,^15,17,28,29^ as shown in Fig. 3d. The crystallinity of MoTe_2_ films improves with increasing annealing, and we do not find any deterioration of MoTe_2_ crystal quality up to 450 °C. Thus, room temperature co-sputtering followed by an *in situ* annealing process can be used to grow 1T′-MoTe_2_ films. The thickness of the MoTe_2_ films can be precisely controlled from an order of a nanometer to tens of nanometers by adjusting the sputtering time.

Fig. 3e shows the temperature dependence of the resistivity of a deposited MoTe_2_ film. Raman spectra in the previous section indicated that the deposited films form in the 1T′ metallic phase. However, the *ρ*–*T* plot shows an increase in resistivity at lower temperatures, which is characteristic of carrier concentrations lower than the metal-insulator transition.^30^ This can be attributed to doping of the material by the V_Te_, consistent with the Te-poor stoichiometry measured with RBS.

### The origin of 1T′-phase stabilization: charge driven 2H → 1T′ phase transition

**Fig. 4:**
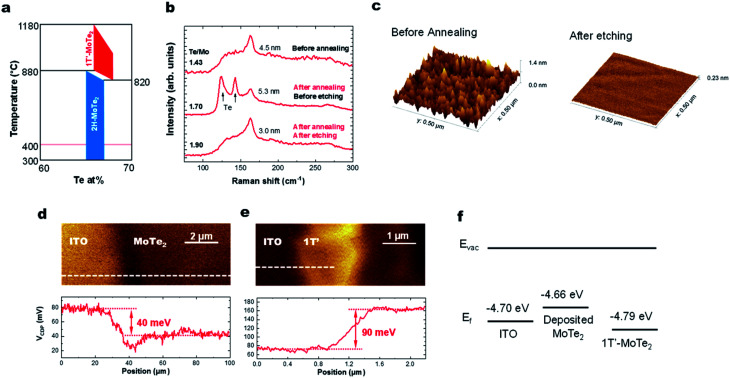
(a) Phase diagram of MoTe_2_. (b) Raman spectra before/after annealing and the etching process. (c) AFM image of the MoTe_2_ films before annealing and after the etching process. KPFM image of (d) deposited MoTe_2_ and (e) exfoliated 1T′-MoTe_2_ crystals. (f) Fermi energies of ITO, deposited MoTe_2_ film and exfoliated MoTe_2_ crystals.

Here we discuss why the high-temperature 1T′ phase forms during the growth reported in this study. As shown by the phase diagram in Fig. 4a, the 2H phase is formed at temperatures lower than that of the 1T′-phase. When the substrate is cooled down to room temperature, the materials would thus be expected to form the 2H-phase alone. However, we observe the presence of only the 1T′-MoTe_2_ phase, presumably as a result of the stabilization of this phase by the presence of the V_Te_ donors.^16,31^ This has previously been documented using ionic gating experiments on exfoliated 2H-MoTe_2_ flakes when the sheet electron density is greater than 2.2 × 10^14^ cm^−2^.^14^ Hall measurements on our MoTe_2_ films show that our sheets contain n-type carriers with a concentration of 4.6 × 10^14^ cm^−2^. Kelvin probe force microscopy (KPFM) data collected from sputtered MoTe_2_ and exfoliated 1T′-MoTe_2_ (Fig. 4d–f) show that the work function of sputtered MoTe_2_ is lower than that of exfoliated 1T′ by 130 meV, indicating that the deposited MoTe_2_ film is decisively n-doped. For a film with a Te/Mo ratio of 1.76, the estimated density of the double-donor V_Te_ per layer is 2.7 × 10^14^ cm^−2^, resulting in a free-electron density of 5.4 × 10^14^ cm^−2^. This is also consistent with recent reports on exfoliated MoTe_2_ sheets that find that a ∼3% V_Te_ concentration is sufficient to stabilize the 1T′-phase over the 2H-MoTe_2_ phase.^1^

### Improving the stoichiometry by high pressure Te annealing

Lastly, we show that the stoichiometry can be further improved using *ex situ* thermal processing. A MoTe_2_ film was deposited at room temperature, *in situ* annealed at 400 °C and then sealed in a glass ampoule with elemental Te and heated at high temperatures (Fig. 1c). A ∼1 torr Te partial pressure in the ampoule was produced by keeping the Te side at 520 °C. The side with the MoTe_2_ film was kept at 250 °C. A thin Te overlayer was formed on the MoTe_2_ film, which was subsequently removed using H_2_SO_4_ (Fig. 4b). During the treatment, excess Mo was removed by H_2_SO_4_ and the Te/Mo ratio of the film increased even though a Te overlayer was removed. After the treatment, the film surface was found to be smoother (Fig. 4c), the MoTe_2_ stoichiometry increased to MoTe_1.90_ and the structure remained in the 1T′ phase, but there was no noticeable change in the Raman FWHM values. Despite the improvement, this value still remains above the 3% vacancy threshold which was previously suggested for stabilizing the 1T′ phase.

## Conclusion

The overall results have established a low temperature soft sputtering technique that can synthesize MoTe_2_ films. The process can produce 2D anisotropic MoTe_2_ 1T′ phase films using room-temperature co-sputtering of Te and Mo under relatively high pressure, followed by *in situ* annealing. This industrially compatible sputtering method offers large area growth capability, thickness control, freedom in substrate selection, and the ability to adjust the stoichiometry. Activation energies for film decomposition were determined by measuring the incorporation rate of Mo and Te into MoTe_2_ films on heated substrates. The crystallization of these MoTe_2_ films required temperatures greater than 300 °C and the deposited MoTe_2_ has always crystallized in the 1T′ anisotropic phase as a result of stabilization by using a high concentration of V_Te_ double-donors. *Ex situ* thermal annealing in a high-pressure Te environment increased the stoichiometry of the 1T′ phase from MoTe_1.86_ to MoTe_1.90_.

## Conflicts of interest

There are no conflicts of interest declared.

## Supplementary Material

NA-002-D0NA00066C-s001
