# Supplementary material for: Low-temperature synthesis of 2D anisotropic MoTe2 using a high-pressure soft sputtering technique
Source: Nanoscale Adv. 2020 Mar 4;2(4):1443–8. doi: 10.1039/d0na00066c (PMC9419816; doi:10.1039/d0na00066c)
Supplement: NA-002-D0NA00066C-s001 [file NA-002-D0NA00066C-s001.pdf]

Electronic Supplementary Material (ESI) for Nanoscale Advances.
This journal is © The Royal Society of Chemistry 2020

Supporting information

Low-temperature synthesis of 2D anisotropic $MoTe_2$ using high-pressure soft sputtering technique

Kentaro Yumigeta[+], *Cameron Kopas*[+], *Mark Blei, Debarati Hajra, Yuxia Shen, Dipesh Trivedi, Pranvera Kolari, Nathan Newman,* and *Sefaattin Tongay*[*]

School for Engineering of Matter, Transport and Energy, Arizona State University, Tempe, AZ 85287, USA

[*] Corresponding author: sefaattintongay@asu.edu

[+] These authors contributed to this work equally

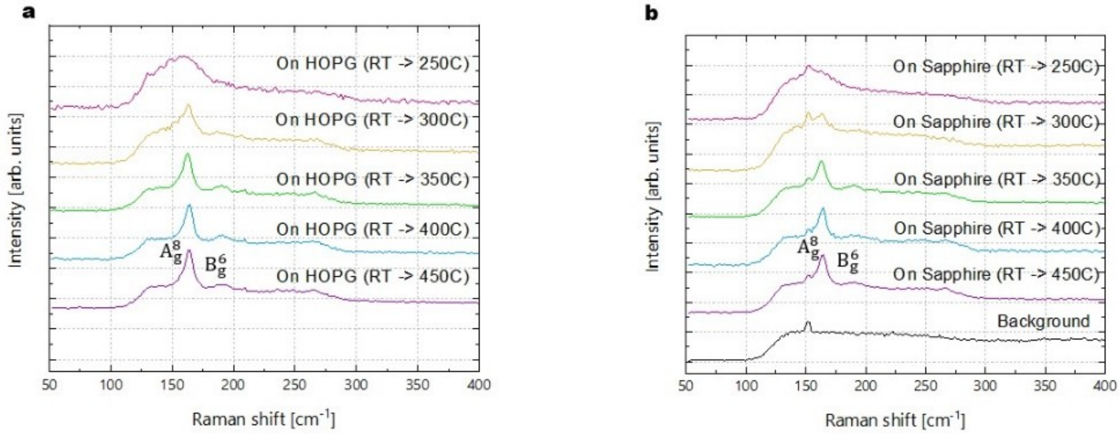

Figure S1 Raman spectra for MoTe2 thin film growth followed by in-situ annealing on **a.** HOPG and **b.** sapphire substrate. Raman spectrum for sapphire substrate is shown as background. Peak at 151 cm^{-1} in background spectrum is from environment lights through transparent sapphire substrate.

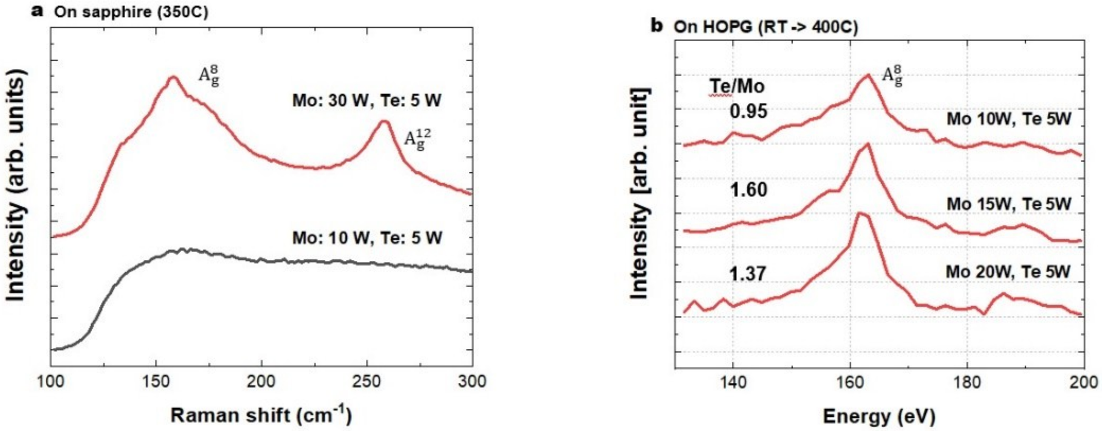

Figure S2 Raman spectra for MoTe2 thin film with different Mo sputtering power **a.** on heated substrates and **b.** room temperature deposition followed by annealing.
